# Lymphokine-activated killer and dendritic cell carriage enhances oncolytic reovirus therapy for ovarian cancer by overcoming antibody neutralization in ascites

**DOI:** 10.1002/ijc.28450

**Published:** 2013-09-18

**Authors:** VA Jennings, EJ Ilett, KJ Scott, EJ West, R Vile, H Pandha, K Harrington, A Young, GD Hall, M Coffey, P Selby, F Errington-Mais, AA Melcher

**Affiliations:** 1 Targeted & Biological Therapies Group, Leeds Institute of Molecular Medicine, University of LeedsUnited Kingdom; 2 Molecular Medicine Program & Department of Immunology, Mayo ClinicRochester, MN; 3 Cardiovascular and Cancer research group, Faculty of Health and Medical Sciences, University of SurreyGuildford, United Kingdom; 4 Division of Cancer Biology, The Institute of Cancer Research, Chester Beatty LaboratoriesLondon, United Kingdom; 5 St. James’s Institute of Oncology, Leeds Teaching Hospitals NHS TrustLeeds, United Kingdom; 6 Oncolytics Biotech IncorporatedCalgary, AB, Canada

**Keywords:** antitumor immunity, malignant ascites, neutralizing antibodies, reovirus

## Abstract

Reovirus is an oncolytic virus (OV), which acts by both direct tumor cell killing and priming of antitumor immunity. A major obstacle for effective oncolytic virotherapy is effective delivery of OV to tumor cells. Ovarian cancer is often confined to the peritoneal cavity and therefore i.p. delivery of reovirus may provide the ideal locoregional delivery, avoiding systemic dissemination. However, ovarian cancer is associated with an accumulation of ascitic fluid, which may interfere with oncolytic viral therapy. Here, we investigated the effect of ascites on reovirus-induced oncolysis against primary ovarian cancer cells and ovarian cancer cell lines. In the absence of ascites, reovirus was cytotoxic against ovarian cancer cells; however, cytotoxicity was abrogated in the presence of ascitic fluid. Neutralizing antibodies (NAb) were identified as the cause of this inhibition. Loading OV onto cell carriers may facilitate virus delivery in the presence of NAb and immune cells which have their own antitumor effector activity are particularly appealing. Immature dendritic cells (iDC), Lymphokine-activated killer (LAK) cells and LAKDC cocultures were tested as potential carriers for reovirus for tumor cell killing and immune cell priming. Reovirus-loaded LAKDC, and to a lesser degree iDC, were able to: (*i*) protect from NAb and hand-off reovirus for tumor cell killing; (*ii*) induce a proinflammatory cytokine milieu (IFNɣ, IL-12, IFNα and TNFα) and (*iii*) generate an innate and specific antitumor adaptive immune response. Hence, LAKDC pulsed with reovirus represent a novel, clinically practical treatment for ovarian cancer to maximise both direct and innate/adaptive immune-mediated tumor cell killing.

**What’s new?:**

Oncolytic viruses (OVs) specifically infect and kill tumor cells. In this study, the authors began to examine whether intraperitoneal delivery of an OV could be effective against ovarian cancer. They found that, while the virus does kill ovarian-cancer cells *in vitro*, this effect is blocked when ascites fluid is added. Cytotoxicity can be restored, however, by using a combination of lymphokine-activated killer and dendritic cells (LAKDC) as carriers, which protect the virus from neutralizing antibodies in the ascites. The LAKDC combination may also support subsequent adaptive immune priming.

Ovarian cancer is the leading cause of death amongst gynaecological malignancies and the fourth most common cause of cancer-related deaths in women, with ∼125,000 deaths per year, worldwide. The existing treatment for epithelial ovarian cancer combines debulking surgery with platinum-based chemotherapy; although initial treatment is generally successful, relapse occurs in the majority of patients, with an overall five-year survival rate for all stages of ∼40%.

Oncolytic viruses (OV) specifically replicate in and lyse tumor cells. In addition to their direct cytotoxicity, OV stimulate the immune system and facilitate the generation of antitumor immune responses by releasing tumor-associated antigens (TAA), danger signals and proinflammatory cytokines into the tumor microenvironment.^1^–^3^ Reovirus is a naturally occurring OV, which is cytotoxic against many tumor types, including melanoma,^4^ breast,^5^ colorectal,^6^ prostate,^2^ ovarian^7^ and head and neck cancer,^8^ and has been investigated in a number of clinical trials.^9^ A major limitation for effective reovirus-based oncolytic virotherapy is the efficient delivery of OV to tumor cells in the presence of intact host immunity. Since ovarian cancer often remains confined to the peritoneal cavity, intraperitoneal (i.p.) administration of OV provides a potential locoregional delivery, direct to the tumor site, as an alternative to intravenous (i.v.) delivery. However, ovarian cancer is often associated with accumulation of ascitic fluid in the peritoneal cavity. Ascites can develop in numerous malignancies, including ovarian, endometrial, colorectal, gastric, breast and pancreatic cancer. At the time of diagnosis, 30% of ovarian cancer patients have developed malignant ascites, which increases to 60% at the time of death.^10^ As malignant ascites arises as a plasma exudate, it contains fractions of protein-rich plasma, including antibodies. Hemminki *et al*. previously reported that neutralizing antibodies (NAb) against adenovirus are found in both the plasma and ascites of ovarian cancer patients.^11^ Since NAb bind viruses, block viral attachment to cell surface receptors and inhibit viral infection, their presence may be detrimental to oncolytic virotherapy. The presence of reovirus specific NAb has been observed in the blood of patients enrolled in reovirus clinical trials^12^–^14^; however, their presence in ascites has yet to be established.

Loading OV onto the surface of cell carriers or using cells which internalize OV has been shown to be an effective mechanism to deliver virus to tumor in the presence of NAb, predominantly in murine models, using carrier cells as “Trojan horses,” which internalize the OV and are permissive for viral replication, but have no subsequent role following OV delivery.^15^–^17^ However, there is variability in terms of viral loading and “hitch-hiking” by carrier cells, including between the mouse and human systems, both in terms of whether the virus replicates in carrier cells during transport and exactly how the virus is protected from NAb. Currently, the precise mechanisms underlying how OV behave on or in their carrier cells en route to their tumor target remain largely undefined. Viral internalization by carrier cells may allow better protection from NAb but will fail if the carrier cell is killed before it reaches the tumor site. In this regard, it is reassuring that reovirus is not toxic to human DC, T cells^18^ or peripheral blood mononuclear cells (PBMC).^19^ Additionally, using immune cells as carriers also has the potential to provide additional antitumor activity, either *via* direct cytotoxicity [*e.g*., natural killer (NK), cytokine-induced killer (CIK) and lymphokine-activated killer (LAK) cells], or by supporting the priming of adaptive antitumor immune responses (*e.g*., DC and macrophages). Combining oncolytic viral therapy with cellular immunotherapy may thus prove to be an effective dual cancer therapy. For example, preclinical studies have demonstrated that vesicular stomatitis virus (VSV) loaded onto tumor-specific T cells and vaccinia virus (VV) loaded onto CIK cells can enhance antitumor therapy compared with either agent alone.^20^,^21^

LAK cells (a heterogeneous population of activated T, NK and NKT cells), which can be easily cultured in large numbers from patients, have shown some promise as a cellular agent for cancer therapy and have been used clinically with IL-2 for the treatment of ovarian cancer.^22^,^23^ LAK cells show specific antitumor activity against autologous ovarian tumor cells and can be expanded *ex vivo* in the presence of IL-2. However, whilst LAK cells alone were well tolerated (up to 10^11^ cells per infusion), concomitant systemic delivery of IL-2 to patients resulted in significant toxicities, including vascular leakage and hypotension. Coculture of LAK cells with DC (LAKDC) has been reported to eliminate the need for coadministration of IL-2, due to bi-directional signaling supporting LAK cell activation and viability, as well as inducing DC maturation and the production of proinflammatory cytokines.^24^–^26^ *In vivo* studies have shown that the combination of LAKDC can effectively eradicate subcutaneous tumors, leading to the generation of antitumor immunity, whereas treatment with either cell type alone was ineffective.^27^ Previous studies have also highlighted DC as effective cell carriers for reovirus in the presence of neutralizing serum for the treatment of melanoma. DC protected reovirus from antibody neutralization by internalization of viral particles, making reovirus unavailable for NAb binding.^18^ The use of DC in combination with LAK cells may, therefore, provide effective cell carriage for reovirus, leading to antitumor responses mediated by direct cytotoxicity and/or the generation of antitumor immunity.

Here, we show that ovarian cancer cell lines and primary ovarian cancer cells established from patients are susceptible to reovirus-induced oncolysis; however, this direct cytotoxicity was abrogated if malignant ascites was present. The inhibitory factor present in the ascites was identified as NAb, which could be found in equal concentrations in both the plasma and ascites of ovarian cancer patients. Loading reovirus onto either immature dendritic cells (iDC) or LAKDC overcame antibody neutralization and reovirus-loaded LAKDC were optimal for delivering reovirus for direct tumor cell killing and innate and adaptive immune priming.

## Material and Methods

### Reovirus

Reovirus Type 3 Dearing strain was provided by Oncolytics Biotech and stored at neat concentrations in PBS at 4°C (up to a month) or at −80°C (long-term storage). Virus titre was determined by standard plaque assays using L929 cells.

### Cell culture

Human cell lines, Skov-3, OVCA433, TR175 and Daudi, were grown in Roswell Park Memorial Institute-1640 medium (RPMI-1640; Sigma) supplemented with 10 % (v/v) fetal calf serum (FCS; Biosera) and 2 mmol/L glutamine (Sigma). L929 and Mel-888 cells were grown in Dulbecco’s Modified Eagle’s Medium (Sigma) supplemented with 10% (v/v) FCS and 2 mmol/L glutamine. PBMC were derived from buffy coats of healthy donors by Ficoll-Hypaque density gradient centrifugation. iDC were generated from monocytes isolated from PBMC using anti-CD14 magnetic beads (Miltenyi Biotec) and cultured in RPMI-1640 supplemented with 10% (v/v) FCS, 2 mmol/L glutamine, 800 U/mL GM-CSF (R&D Systems) and 500 U/mL rhIL-4 (R&D Systems) for 7 days. LAK cells were generated from the CD14 negative fraction of PBMC by culturing in RPMI-1640 supplemented with 10% (v/v) FCS, 2 mmol/L glutamine and 500 U/mL rhIL-2 (Proleukin®) for 7 days.

### Ovarian cancer samples

Ovarian cancer patients (all serous adenocarcinomas, Stages III–IV) undergoing routine paracentesis were identified and informed consent was given to collect ascitic fluid in accordance with local institutional ethics review and approval. Ascitic fluid was centrifuged at 450*g* for 10 min; fluid was collected and stored at 4°C or −20°C for long term storage. Primary ascites-derived cancer cells were cultured in RPMI-1640 containing 10% (v/v) FCS, 7.5% (v/v) autologous ascitic fluid and 2 mmol/L glutamine. Tumor cells were identified by positive staining of CA125 (GeneTex) using flow cytometry and used at low passage numbers (p1-p4).

### Reovirus infection of ovarian cancer cells

Ovarian cancer cell lines and ascites-derived cancer cells were seeded at 1 × 10^5^ in the presence or absence of 2.5% (v/v) ascitic fluid. Matched autologous cells and ascites were available for four patient samples. Reovirus was added to adherent cells at indicated concentrations. Cells were then cultured for denoted times before viability was determined.

### Live/dead® viability assay

Cell viability was determined using the Live/dead® viability assay (Life Technologies) following manufacturer’s instructions. Cells were acquired on a FACS Calibur (Becton-Dickinson) and percentage of cell death determined.

### Ascites and plasma neutralization

L929 cells were seeded into a flat bottomed 96-well plate at a cell density of 2.5 × 10^4^ cells/well. Halving dilutions of plasma and ascites samples were prepared before the medium was removed from L929 cells and replaced with the plasma- and ascites-diluted medium. Cells were infected with reovirus at 0.05 pfu/cell or left uninfected for 100% viable control. After 48 hr, MTT (3-(4,5-dimethylthiazol-2-yl)-2, 5-diphenyltetrazolium bromide) (5 mg/mL; Sigma) was added to wells and incubated for 4 hr at 37°C. Medium was removed and cells solubilized using 100% DMSO (Fisher Scientific). Optical density was determined using a Multiskan EX plate reader (Thermo Electron) at a wavelength of 550 nm.

### Removal of human IgG from ascites

Protein A Resin-Higher Binding Capacity (GenScript) was used to isolate IgG from the ascites, following manufacturer’s instructions.

### Immunoprecipitation of reovirus from ascites

Reovirus (5 × 10^5^ pfu) was incubated in ascites for 30 min, before the addition of protein A resin. Resin and ascites were incubated for 2 hr at 4°C; IgG was then removed as above. The IgG samples were then analyzed by standard western blotting for reovirus σ3 protein (clone 4F2; DSHB).

### Reovirus loading of carrier cells

Carrier cells (iDC and LAK cells) were resuspended at 5 × 10^6^ cell/mL in PBS; LAKDC cocultures were set up at a 1:1 ratio, using 5 × 10^6^ cells/mL of each cell type. Cells were incubated ± reovirus for 3–4 hr at 4°C; the amount of reovirus added was the same in all 3 cultures: 1 pfu/cell for single cell cultures and 0.5 pfu/cell in LAKDC cocultures. Reovirus-loaded cells were washed twice with PBS to remove any unbound reovirus.

### Reovirus retention and replication

Reovirus-infected and -loaded cells were collected at indicated time points and subjected to three cycles of freeze-thaw lysis. Reovirus retention and replication was determined by standard plaque assay on L929 cells.

### Reovirus hand-off in the presence of ascites

Reovirus-loaded iDC, LAK and LAKDC were added to adherent TR175 cells at a 1:1 ratio ± 2.5% ascitic fluid. After 4 hr, medium containing carrier cells was removed and replaced with medium ± 2.5% ascites. TR175 cells were cultured for a further 72 hr and viability determined by Live/dead® assay.

### Chromium release assay

Cytotoxicity was measured using chromium release assays. Skov-3, OVCA433 and Daudi targets were incubated with 100 µCi ^51^Cr (Perkin Elmer) for 1 hr, washed three times with a large volume of Hanks Balanced Salt Solution (HBSS; Sigma) and incubated with effectors at indicated ratios. ^51^Chromium release was determined using a 1450 MicrobetJet Scintillation Counter (Perkin Elmer) and the results were expressed as percent specific target lysis, using the formula:
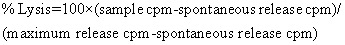


### Flow cytometry

A FACS Calibur was used for acquisition and analysis was performed using Cell Quest Software, v4.0.1 (BD Biosciences). Antibodies: antihuman CD80-PE, CD86-PE, CD11c-FITC, CD107a-FITC, CD107b-FITC and CD8-PerCP (all BD Pharmingen).

### ELISA

Human IFNɣ, TNFα, IL-12p70 (BD Pharmingen), IFNα and IgG (both Mabtech) ELISA assays were carried out using matched antibody pairs, according to the manufacturer’s instructions.

### Generation of tumor specific cytotoxic T cells (CTL)

Reovirus (1 pfu/cell) or iDC, LAK and LAKDC loaded with reovirus (1 pfu/cell) were cultured with Skov-3 cells (1:1 ratio) for 24 hr. Nonadherent cells were harvested and co-cultured with autologous PMBC at a ratio of 1:10 to 1:40 in CTL medium [RPMI-1640 supplemented with 7.5% (v/v) human AB serum (Biosera), 2 mmol/L glutamine, 1% (v/v) sodium pyruvate, 1% (v/v) non-essential amino acids, 1% (v/v) Hepes, 20 μmol/L β-mercaptoethanol (all Sigma)] and 5 ng/mL rhIL-7 (R&D Systems). Cultures were re-stimulated weekly using the same protocol.

### CD107 degranulation assay

CTL and tumor targets were incubated at a 1:1 ratio; after an hour anti-CD107a- and CD107b-FITC, anti-CD8-PerCP and Brefeldin A (10 μg/mL; Sigma) was added. After a further 4 hr, CTL were washed and analyzed by flow cytometry.

### Statistics

One-way ANOVA was used to determine statistical significance.

## Results

### Ascites inhibits reovirus-induced oncolysis and replication

**Figure 1:**
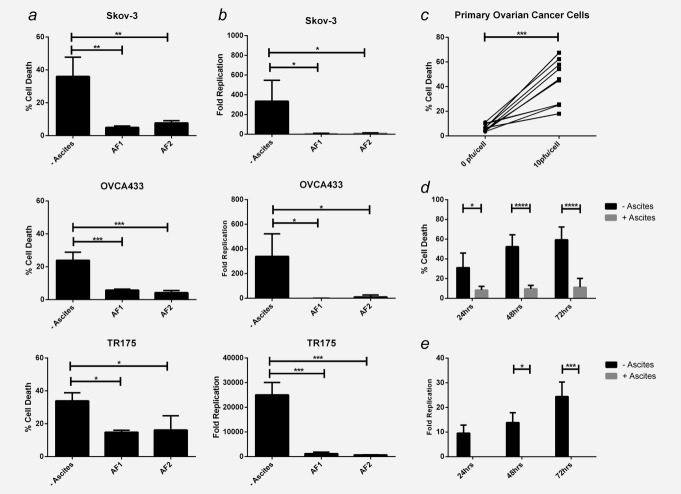
Malignant ascites inhibits reovirus-induced oncolysis. Ovarian cancer cell lines, Skov-3, OVCA433 and TR175 were infected with reovirus (1 pfu/cell) ± 2.5% ascitic fluid (2 separate samples; AF1 and 2). (*a*) Cell viability was determined using the Live/dead® viability assay and flow cytometry 48 hr postinfection (p.i.). Graphs show the mean percentage cell death + SEM from three independent experiments. (*b*) Reovirus replication was determined by plaque assay using L929 cells and fold increase was calculated from initial input reovirus. Graphs show mean fold increase of reovirus titres + SEM at 72 hr p.i. from three independent experiments. (*c*) Primary ascites-derived ovarian cancer cells from 10 patients were infected ± reovirus (10 pfu/cell) and cell viability was determined 48 hr p.i. using the Live/dead® assay. (*d*) Four primary samples were infected with reovirus (10 pfu/cell) ± 2.5% autologous ascitic fluid (samples AF3, 4, 5 and 6) for 24, 48 and 72 hr and cell viability determined as above. Graph shows the mean percentage cell death + SEM from four patient samples. (*e*) Reovirus replication was determined in primary ascites-derived ovarian cancer cells. Cells were infected with reovirus (1 pfu/cell) for 24, 48 and 72 hr ± 2.5% autologous ascitic fluid (samples AF3, 4 and 7) and reovirus replication determined as in (*b*). Graph shows the mean + SEM from three patient samples (*p < 0.05, **p < 0.01, ***p < 0.001 and ****p < 0.0001).

Whilst reovirus-induced oncolysis of ovarian cancer cell lines has been previously demonstrated,^7^ the effects of reovirus-induced oncolysis on primary human ovarian cancer cells and its effects in the context of ascitic fluid have not been examined. In the absence of ascites, reovirus was cytotoxic against three established ovarian cancer cell lines, Skov-3, OVCA433 and TR175 (Fig. 1*a*) and, importantly, against 10 primary ovarian cancer cell samples freshly isolated from patients’ ascites, albeit with varying sensitivities (Fig. 1*c*). However, in the presence of 2.5 % ascitic fluid, reovirus-induced cytotoxicity was significantly inhibited in all cell lines and in four out of four patient tumor samples cultured in autologous ascites (Figs. 1*a* and 1*d*). Reovirus replication was also examined following infection of ovarian cancer cells ± ascites. In the absence of ascites, reovirus readily replicated in ovarian cancer cell lines (Fig. 1*b*) and primary ovarian cancer cells (Fig. 1*e*, although at 10- to 1000-fold lower levels than those detected in cell lines). However, in the presence of ascites, reovirus replication, in line with tumor cell killing, was significantly abrogated (Figs. 1*b* and 1*e*).

### Antibodies present in ascites are responsible for blocking reovirus-induced oncolysis

**Figure 2:**
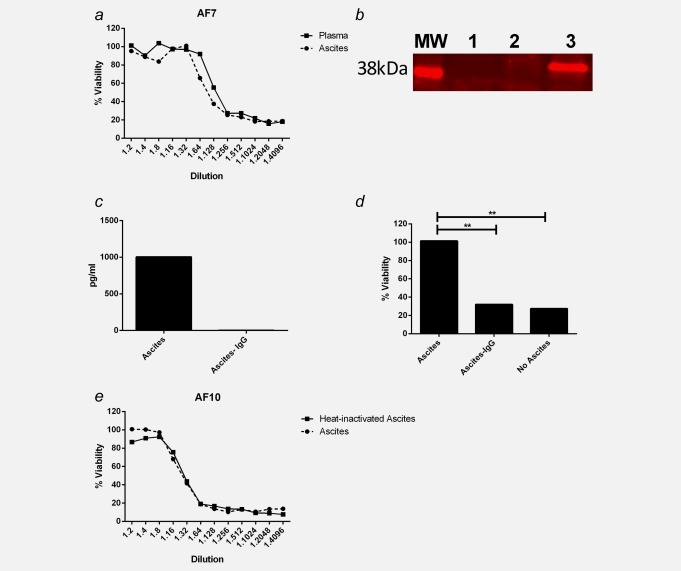
Reovirus inhibition is IgG-mediated. (*a*) L929 cells were infected with reovirus (0.05 pfu/cell) for 48 hr in the presence of serial dilutions of matched patient plasma and ascites (AF7). L929 cell viability was determined by MTT assay and normalized to uninfected control. Graph is representative of five patient samples. (*b*) IgG was immunoprecipitated from ascites, which had been incubated ± reovirus (5 × 10^6^ pfu) using protein A resin, then probed for reovirus σ3 protein (41 kDa MW) by western blotting. MW: molecular weight marker; Lane1: ascites (AF3) + protein A resin (no reovirus); Lane 2: protein A resin + reovirus [no ascites (IgG)]; Lane 3: ascites (AF3) + reovirus + protein A resin. Blot is representative of six ascitic samples. IgG-depleted ascites samples were collected by centrifugation following protein A resin incubation. (*c*) IgG concentration in ascites before and after IgG depletion was determined by ELISA. (*d*) L929 cells were infected ± reovirus (0.05 pfu) either in the presence of ascites, IgG-depleted ascites or in the absence of ascites, for 48 hr. Cell viability was determined by MTT assay and normalized to uninfected control. Graphs shows representative ascitic sample, AF9, of three samples (AF1, 2 and 9). (*e*) L929 cells were infected with reovirus (0.05 pfu/cell) for 48 hr in the presence of serial dilutions of matched ascites ± heat inactivation (AF10). L929 cell viability was determined by MTT assay and normalized to uninfected control. Graph is representative of three patient samples (**p < 0.01).

To determine whether the inhibition of reovirus was ascites-specific or systemic, matched plasma and ascites samples from ovarian cancer patients were analyzed. Both plasma and ascites inhibited reovirus-induced cytotoxicity to a similar level, as seen by neutralization of L929 cell death (Fig. 2*a*). These data suggest a systemic inhibitory factor was responsible; hence, we hypothesized that NAb may play a role in reovirus inhibition. To establish whether reovirus was directly bound to NAb in the ascites, immunoprecipitation assays were performed. Reovirus σ3 capsid protein was successfully immunoprecipitated with IgG in six ascites samples tested; a representative blot is shown in Figure 2*b*. Furthermore, removing IgG from the ascites, confirmed by ELISA (Fig. 2*c*), significantly restored reovirus-induced cytotoxicity (Fig. 2*d*), indicating a major role for IgG inhibiting reovirus-induced cell death in ascites. To determine whether complement proteins were also involved in the inhibition of reovirus, ascites samples were heated to 56°C for 30 min to inactivate complement before neutralization assays were performed. There was no difference observed in reovirus-induced cytotoxicity when comparing heat-inactivated ascites to nonheat-inactivated ascites (Fig. 2*e*), or heat inactivated plasma versus nonheat inactivated plasma (data not shown), confirming that complement was not involved in the inhibition of reovirus.

### iDC and LAKDC deliver reovirus to tumor cells in the presence of neutralizing ascites

**Figure 3:**
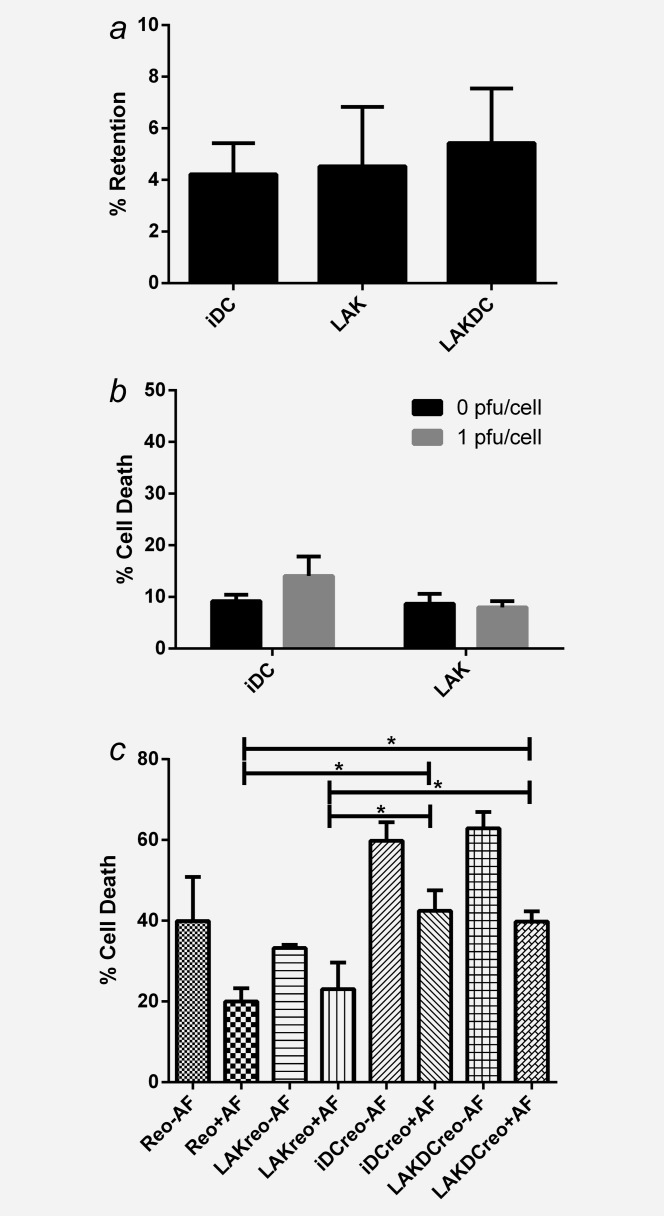
Reovirus-loaded iDC and LAKDC can deliver reovirus for tumor cell killing in the presence of ascites. Reovirus was loaded onto iDC, LAK cells and LAKDC cocultures (1pfu/cell) and either quantified for: (*a*) Percentage of reovirus retention by plaque assay or (*b*) Cytotoxicity by Live/dead® viability assay at 48 hr postloading. Graphs show the mean + SEM of three healthy donors. (*c*) TR175 were treated with neat reovirus or reovirus-loaded onto iDC, LAK cells and LAKDC (iDC-reo, LAK-reo and LAKDC-reo) for 4 hr ± 2.5% ascites. Carrier cells were then removed and replaced with growth media ± ascites; TR175 cell viability was then determined 72 hr p.i. by Live/dead® viability assay. Graph shows mean percentage of cell death + SEM from five healthy donors using two ascitic fluids (AF1 and 7; *p < 0.05).

Whilst reovirus has previously been successfully loaded onto human iDC for tumor delivery,^18^ the ability of LAK cells or LAKDC cocultures to carry and protect reovirus from NAb, has not been investigated. To first identify their suitability as potential viral carriers, iDC, LAK cells and LAKDC cocultures were loaded with reovirus and their levels of reovirus retention and replication determined by plaque assay. Approximately 5% of input virus was retained by all cell populations (Fig. 3*a*) and no reovirus replication was detected up to 72 hr postloading (data not shown). In addition, for iDC and LAK cells to be effective cell carriers and provide additional antitumor effects, it was important to establish that they remained viable following reovirus-loading. Viability studies showed no significant increase in death 48 hr post-reovirus loading (Fig. 3*b*). Next, the ability of iDC, LAK cells and LAKDC cocultures to deliver reovirus to tumor cells in the presence of NAb was investigated. Neat reovirus and reovirus-loaded iDC, LAK and LAKDC (iDC-reo, LAK-reo and LAKDC-reo) were cultured with TR175 cells at a 1:1 ratio for 4 hr ± 2.5% ascitic fluid. Carrier cells were removed to exclude cell-mediated TR175 cytotoxicity (no TR175 cell death was detected at 4 hr at this E:T ratio – data not shown), and tumor cells were cultured (± 2.5% ascitic fluid) for a further 72 hr to investigate reovirus-induced cytotoxicity. Figure 3*c* shows that levels of tumor cell death in the presence of ascites following iDC-reo and LAKDC-reo viral hand-off were significantly greater than for neat reovirus or LAK-reo delivery. These data indicate that iDC or LAKDC cocultures are effective cell carriers for delivering reovirus to tumor cells in the presence of NAb.

### Reovirus-loaded LAKDC induce ovarian cancer cell death

**Figure 4:**
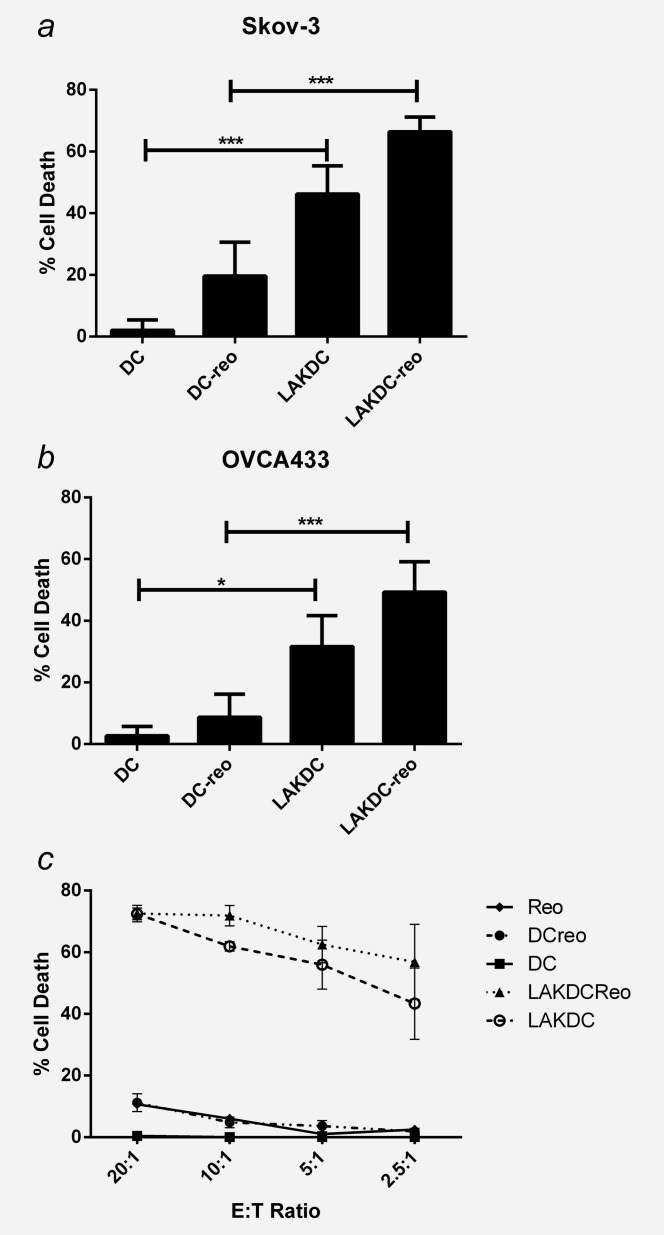
Reovirus-loaded LAKDC are more cytotoxic towards ovarian cancer cells than reovirus-loaded iDC. Cytotoxicity of reovirus-loaded iDC and LAKDC was determined by chromium release assay. (*a*) Skov-3 and (*b*) OVCA433 targets were labeled with chromium and cultured with iDC-reo or LAKDC-reo in the presence of 2.5% ascites (AF11, 12 and 13) at indicated E:T ratios for 48 hr. Graphs show the mean percentage cell death + SEM from three healthy donors. (*c*) Daudi cells were labeled with chromium and incubated with neat reovirus (1 pfu/cell), reovirus-loaded or -unloaded iDC and LAKDC at indicated E:T ratios for 48 hr. Graph shows the mean percentage cell death ± SEM from three healthy donors (*p < 0.05, ***p < 0.001).

Having determined that iDC and LAKDC have the potential to deliver oncolytic reovirus to tumors in the presence of NAb to induce virus-mediated cytotoxicity, we next investigated whether these carrier cells could provide additional immune-mediated antitumor effects. As reovirus-loaded LAK cells were unable to shield the virus from neutralization, they were not investigated further (Fig. 3*c*). To determine the cytotoxic potential of iDC and LAKDC cell carriers alone ± loading with reovirus, in the presence of ascites, chromium release assays were performed. LAKDC, unlike iDC, were cytotoxic against Skov-3 and OVCA433 cells and loading reovirus onto either LAKDC or iDC further increased tumor cell death (Fig. 4*a* and 4*b*). We reasoned this additional cytotoxicity following the loading of reovirus could be either direct viral cytotoxicity, or related to an alternative indirect immune mechanism whereby reovirus augments the cytotoxic effects of NK cells.^28^ To explore these alternatives, the ability of reovirus to enhance LAKDC-induced cytotoxicity was investigated using Daudi as a target cell line, which is known to be resistant to direct reovirus oncolysis. LAKDC-reo were able to kill Daudi targets, but there was no difference between LAKDC and LAKDC-reo cytotoxicity (Fig. 4*c*), implying that reovirus did not enhance immune LAKDC-mediated cytotoxicity; rather, any increased killing by LAKDC-reo over LAKDC in Figure 4*a* was probably due to viral, rather than immune effects. Similarly, additional DC-reo cytotoxicity against Daudi targets was not seen (Fig. 4*c*). Overall, these data support the potential use of LAKDC as carriers of reovirus *in vivo* for ovarian cancer, to instigate innate immune-mediated cytotoxicity in the presence of NAb, together with reovirus-induced oncolysis.

### LAK cells and reovirus can mature iDC in the presence of ascites

**Figure 5:**
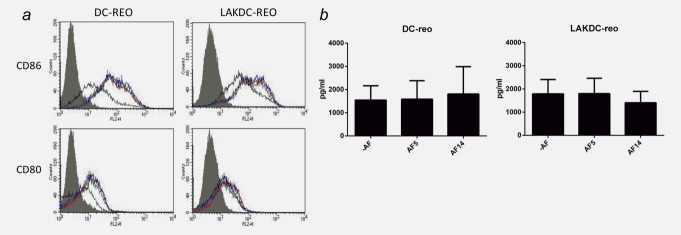
LAKDC and reovirus phenotypically mature iDC and produce proinflammatory cytokines in the presence of ascites. (*a*) Reovirus-loaded iDC or LAKDC were cultured in the absence (red line) or presence of 2.5% AF5 (green line) or AF14 (blue line) for 48 hr. DC (CD11c+ cells) were analyzed by flow cytometry for expression of maturation/activation markers, CD80 and CD86. Histogram plots are representative of two healthy donors. Shaded gray = isotype control, black line = unloaded DC controls [either iDC (left panel) or DC within LAKDC coculture (right panel)]. (*b*) Reovirus-loaded iDC and LAKDC were cultured for 48 hr ± ascites (AF5 and 14) before supernatants were collected and concentrations of IFNα were determined by ELISA. Graphs show the mean + SEM of four healthy donors.

Previous studies have demonstrated the ability of both LAK cells and reovirus to mature iDC, an essential step for generation of antigen-specific adaptive T cell antitumor immunity.^25^,^28^ However, whether this applies in the context of malignant ascites, which may contain immunosuppressive factors that might impede DC maturation, has not been addressed. The ability of LAK cells and reovirus to phenotypically mature iDC in the presence of ascites was investigated by flow cytometry. Unloaded LAK cells induced cell surface expression of the activation/maturation markers, CD86 and CD80, on iDC to the same extent in both the absence and presence of ascitic fluid (Supporting Information Fig. S1). CD86 was further increased in reovirus-loaded LAKDC cocultures and, again, this was not impeded by the presence of ascitic fluid (Fig. 5*a*), suggesting that both LAK and reovirus can overcome potentially suppressive factors in the ascites.

### Reovirus-loaded LAKDC induce a proinflammatory cytokine milieu

A key determinant of adaptive immune priming following DC maturation is the profile of appropriate cytokines (including TNFα, IFNα, IL-12p70 and IFNɣ) secreted within the tumor microenvironment. In the absence of ascites, loading reovirus onto both iDC and LAKDC significantly induced the secretion of TNFα and IFNα, whilst the production of IL-12p70 and IFNɣ was increased by reovirus in LAKDC-reo cultures compared with LAKDC alone [only low levels of cytokines were detected on reovirus loading of iDC alone (Supporting Information Fig. S2)]. Hence, LAKDC-reo may have the potential to reverse the immunosuppressive tumor microenvironment and promote priming of adaptive antitumor immunity following tumor cell death. To determine whether the production of IFNα, a key cytokine in reovirus-mediated immune cell cross-talk,^19^ which is also central to adaptive immune priming,^29^ was affected in the presence of ascites, levels were measured in iDC-reo and LAKDC-reo cultures ± ascites (iDC and LAKDC do not produce IFNα in the absence of reovirus–data not shown). Figure 5*b* shows that the presence of ascitic fluid did not significantly inhibit the production of IFNα from either LAKDC-reo or DC-reo.

### Reovirus-loaded LAKDC prime antitumor immunity in the presence of malignant ascites

**Figure 6:**
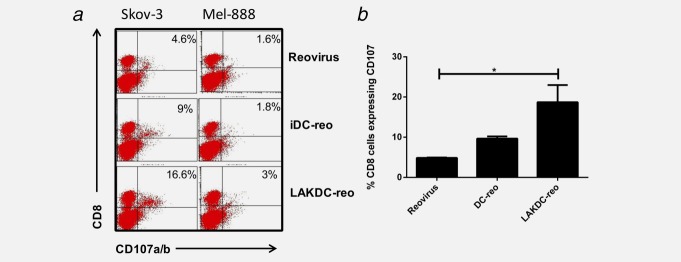
Reovirus-loaded LAKDC prime antitumor immunity in the presence of ascites. Skov-3 cells were infected with either reovirus, iDC-reo or LAKDC-reo in the presence of 2.5% ascites (AF4) for 24 hr. Nonadherent carrier cells/dead tumor cells were removed and cultured with autologous PBMC for a week. CTL were restimulated weekly and tumor specific CTL activity measured in a CD107 assay against specific Skov-3 targets or irrelevant Mel-888 targets. (*a*) FACS plots are representative of three donors and the percentage of CD8 cells degranulating against targets is shown. (*b*) Graph shows the mean percentage + SEM of CD8 cells degranulating against Skov-3 targets from three healthy donors (*p < 0.05).

Finally, to test the ability of reovirus or reovirus-loaded cells to generate a tumor specific adaptive immune response in the presence of ascites, an *in vitro* priming assay was performed. Skov-3 cells were cultured with reovirus, iDC-reo or LAKDC-reo for 24 hr, after which the nonadherent cells were harvested [containing antigen-loaded antigen presenting cells (APC)[ and cultured with autologous PBMC. CTL were restimulated up to twice more. One week after the final restimulation, tumor-specific cytotoxic activity was measured using CD107 degranulation assays against Skov-3 and irrelevant Mel-888 targets. Both neat reovirus and iDC-reo generated some CTL responses, reflected by low level specific CD8 degranulation against Skov-3 cells (Fig. 6*a*); however, the highest levels of adaptive response were seen after priming initiated by LAKDC-reo. This is consistent with the ability of LAKDC-reo to kill tumor cells for antigen release in an inflammatory milieu, *via* both direct oncolysis and innate immune mechanisms, even in the presence of ascitic NAb. Over multiple experiments, LAKDC-reo generated significantly higher proportions of anti-Skov-3 CTL responses than neat reovirus, although the superiority of LAKDC-reo over iDC-reo did not reach statistical significance (Fig. 6*b*). The ability of LAKDC-reo to generate the highest proportion of anti-Skov-3 CTL may be due to greater killing for antigen release (Fig. 4) and/or the enhanced production of pro-inflammatory cytokines (Supporting Information Fig. 2). Taken together, these data suggest that LAKDC are consummate cell carriers for reovirus, allowing delivery of reovirus for tumor cell oncolysis, additional innate immune-mediated killing, production of proinflammatory cytokines and maturation of iDC to support adaptive antitumor immune priming.

## Discussion

Ovarian cancer remains a disease with a poor prognosis, despite initial sensitivity to chemotherapy. Reovirus is one of a range of OV which is being tested.^9^ Although we have shown in patients that i.v. “neat” reovirus is able to selectively access tumors in patients by evading NAb *via* hitchhiking on cells in the blood,^30^ the delivery and efficacy of OV may be improved if they are first pulsed *ex vivo* into carrier cells to enhance OV protection and/or provide additional immune-mediated antitumor cytotoxicity. In the case of ovarian cancer, OV can be delivered by i.v. or i.p. routes (both are currently being tested clinically in reovirus trials); however, regardless of the administration technique, once the virus reaches the tumor microenvironment (often restricted to the peritoneum in ovarian cancer), it will inevitably encounter potentially inhibitory factors. In the case of advanced ovarian cancer, the associated ascitic fluid is known to contain immunosuppressive cytokines, such as IL-10,^31^ TGF-β^32^,^33^ and VEGF,^33^ which could impair the immune-mediated component of OV therapy and/or the effector function of virus-loaded immune cell carriers. Moreover, the direct oncolytic effect of OV against ovarian cancer could be compromised by NAb or other antiviral components within ascites.

In this study, we have shown that ovarian cancer cell lines and primary ovarian cancer cells are sensitive to reovirus-induced oncolysis; however, this susceptibility is abrogated when malignant ascitic fluid is present (Fig. 1). We hypothesized that NAb, which are present at baseline in the blood and rise upon administration of reovirus in cancer patients, were present in the ascites of ovarian cancer patients and responsible for inhibition of oncolysis. We found that matched plasma and ascites samples similarly inhibited reovirus-induced cytotoxicity (Fig. 2*a*), suggesting that a common factor was indeed responsible for reovirus inhibition. The presence of antireoviral antibodies in ascites was confirmed by immunopreciptation of reovirus with IgG (Fig. 2*b*), and depletion of IgG fully restored reovirus-induced oncolysis, confirming IgG was responsible for viral inhibition (Figs. 2*c* and 2*d*). Heat inactivation of ascites/plasma did not affect the ability of ascites/plasma to reduce oncolysis (Fig. 2*e* and data not shown), indicating that complement proteins do not play a role in reovirus inhibition. These findings are consistent with previous data demonstrating that removal of IgG from ascites improves adenoviral infection.^11^,^34^ Hence, strategies that prevent neutralization of reovirus are important for enhancing viral delivery to tumor cells and direct cell killing.

Previous studies, from our own and other laboratories, have shown that OV can be delivered to tumors in the presence of neutralizing serum *via* immune cell carriers,^15^,^21^ and that internalization of the virus may be the mechanism by which OV are protected from NAb.^18^ As immune cells can have direct antitumor effects, using them as carriers of OV could provide additional therapy. We have shown that DC are effective cell carriers of reovirus for the treatment of melanoma,^18^ and there is evidence that the addition of LAK cells to DC may generate a clinically practical and effective cellular immunotherapy.^27^ We, therefore, hypothesized that LAKDC co-cultures could be used as carriers to deliver reovirus to ovarian cancer to: (*i*) protect from NAb in the circulation and/or the tumor microenvironment for delivery to target malignant cells; (*ii*) provide additional early innate tumor cell killing *via* LAK cells and (*iii*) support priming of adaptive antitumor immunity *via* DC.

In this study, we have focused on preclinical human *in vitro* model systems, including the use of fresh patient samples comprising tumor cells and ascites. We have not used murine models because there are significant differences between the effects of reovirus in mouse and human systems; for example, human DC protect reovirus from NAb by internalizing the virus without cell toxicity or viral replication, whilst murine DC support reovirus replication and die after infection.^15^,^18^ Moreover, human data are more directly relevant to clinical application, and we have previously shown LAKDC represent a practical immunogenic mixed cell population, which can readily be prepared to clinical grade from patient blood.^25^

Reovirus could be successfully loaded on/in to iDC, LAK cells and LAKDC cocultures without affecting the cells’ viability, and iDC and LAKDC could protect and hand-off reovirus to tumor cells in the presence of neutralizing ascites, whilst LAK cells and neat reovirus could not (Fig. 3). The mechanism(s) by which DC release reovirus following protective internalization^18^ remain unclear. Another RNA virus (HIV-1) can be transmitted from DC to T cells *via* exocytosis of exosomes,^35^ whilst reovirus can be released from endothelial cells without associated apoptosis or lysis.^36^ Reovirus virions have also been reported to localize to recycling endosomes, suggesting a further mechanism by which the virus may be released.^37^ However, how reovirus is delivered from carrier to target tumor cells to initiate oncolysis remains an important area of future study. Both LAKDC and LAKDC-reo were more cytotoxic against ovarian targets than iDC and iDC-reo, respectively, confirming additional innate immune killing in the presence of LAK cells only (Fig. 4). The increased cytotoxicity following reovirus loading of either LAKDC or iDC populations was probably due to viral-, rather than immune-, mediated cell death, since enhanced cytotoxicity was lost when reovirus-resistant Daudi cells were used as targets. LAK cells and reovirus phenotypically matured iDC in the presence of ascites (Supporting Information Fig. S1 and S5*a*), and hence, may be able to mature suppressed resident iDC (unpublished data). Loading reovirus into LAKDC cocultures also increased production of the immunostimulatory cytokines, IFNɣ, TNFα, IFNα and IL-12p70, potentially reversing the suppressive nature of the tumor milieu to support generation of effective T cell responses (Supporting Information Fig. S2).

As the presence of intra-tumoral T cells in ovarian and other cancer patients has been shown to result in improved survival,^38^ we also tested whether reovirus-loaded carrier cells could support generation of CTL against ovarian cancer, using our previously established model of human *in vitro* priming of antitumor adaptive immunity.^39^ In the presence of ascites, LAKDC-reo were most effective at generating anti-Skov-3 CTL, and were significantly better than neat reovirus (Fig. 6).

In summary, LAKDC-reo may represent a clinically practical therapy, which has the potential to be both an effective direct cytotoxic and innate/adaptive immunotherapy for ovarian cancer, even in the presence of ascites, which contains antiviral NAb. Clinical studies are warranted and in development to test this approach further in patients by administering LAKDC-reo *via* i.v. and i.p. routes.

## Abbreviations

AF: ascitic fluid
APC: antigen presenting cells
CIK: cytokine-induced killer
DC: dendritic cells
i.p.: intraperitoneal
i.v.: intravenous
iDC: immature DC
LAK: lymphokine-activated killer
NAb: neutralizing antibodies
NK: natural killer
NKT: natural killer T
OV: oncolytic viruses
p.i.: post-infection
PBMC: peripheral blood mononuclear cells
pfu: plaque-forming unit
TAA: tumor-associated antigens
VSV: vesicular stomatitis virus
VV: vaccinia virus
